# Supplementary material for: New Sustainable Process for Hesperidin Isolation and Anti-Ageing Effects of Hesperidin Nanocrystals
Source: Molecules. 2020 Oct 3;25(19):4534. doi: 10.3390/molecules25194534 (PMC7582684; doi:10.3390/molecules25194534)
Supplement: Supplementary file 1 [file molecules-25-04534-s001.pdf]

Supplementary Materials

New Sustainable Process for Hesperidin Isolation and Anti-Ageing Effects of Hesperidin Nanocrystals

Danijela Stanisic [1], Leticia H. B. Liu [1], Roney V. dos Santos [1], Amanda F. Costa [1], Nelson Durán [2,3] and Ljubica Tasic [1,*]

[1] Biological Chemistry Laboratory, Organic Chemistry Department, Institute of Chemistry, University of Campinas (UNICAMP), 13083-970, Campinas, SP, Brazil; dacici.stanisic@gmail.com (D.S.); leticia.bacellar@gmail.com (L.H.B.L.); torroney@gmail.com (R.V.d.S.); amanda.fc92@gmail.com (A.F.C.)

[2] Laboratory of Urogenital Carcinogenesis and Immunotherapy, Department of Structural and Functional Biology, University of Campinas, 13083-862, Campinas, SP, Brazil; nelsonduran1942@gmail.com

[3] Nanomedicine Research Unit (Nanomed), Federal University of ABC (UFABC), 09210-580, Santo André, SP, Brazil.

* Correspondence: ljubica@unicamp.br; Tel.: +55-19-3521-1106

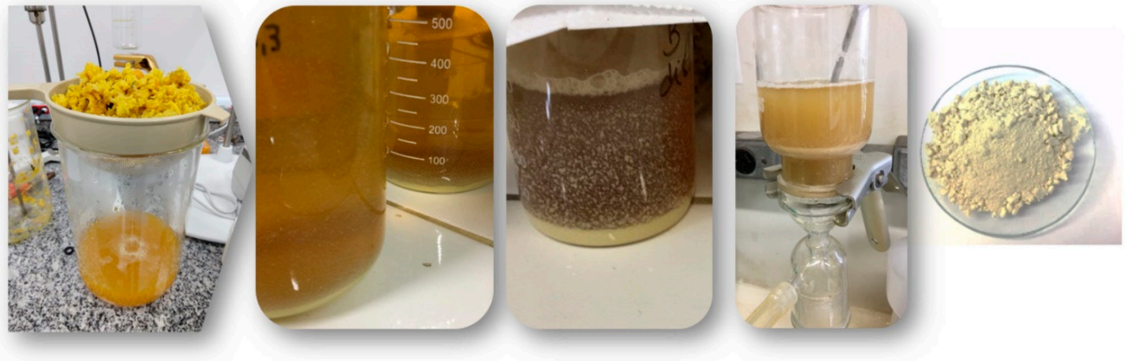

(a) **(b)** **(c)** **(d)** **(e)**

Figure S1. Steps used for hesperidin water extraction: (a) water extract obtained from orange peel (alkaline solution); (b) hesperidin precipitation after neutralisation and low pH; (c) precipitated hesperidin (4 °C); (d) filtration of precipitated hesperidin; (e) dried yellow powder of hesperidin.

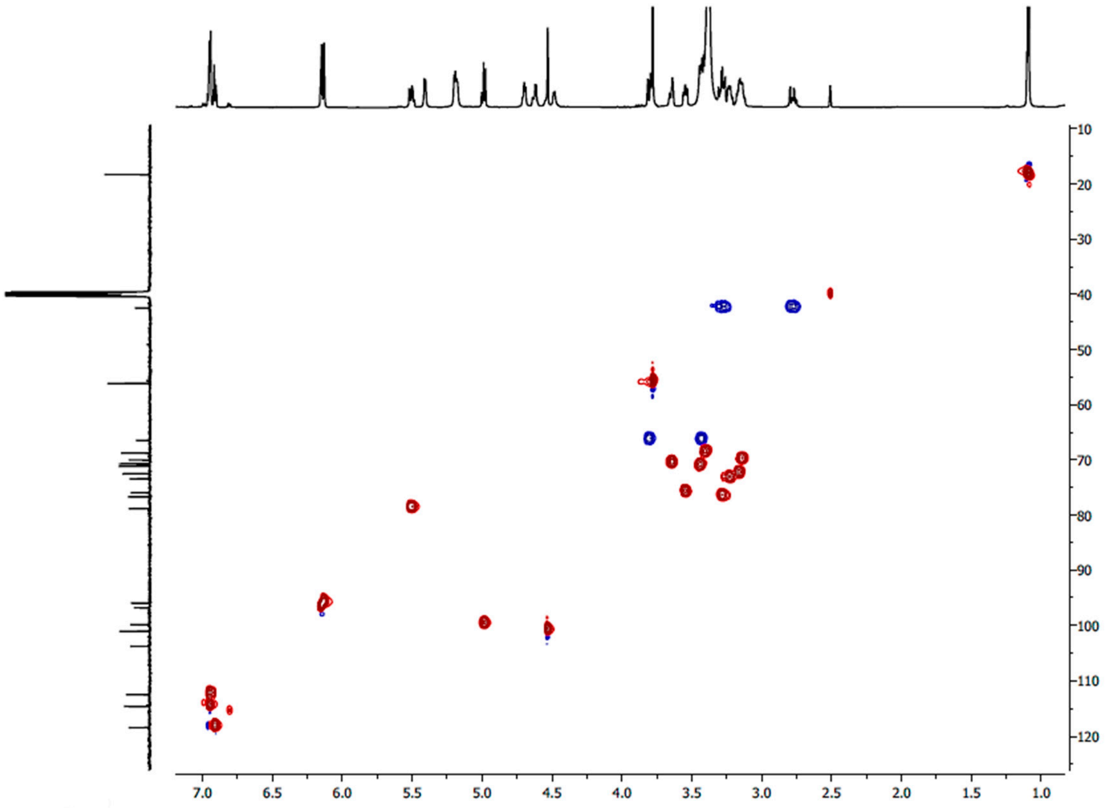

Figure S2. HSQC NMR data of obtained hesperidin (20 mg mL^{-1}) in DMSO-d_6 (2.50 ppm; 39.50 ppm) as a solvent, on Bruker *AVANCE* III 600 MHz equipment at 25 °C.

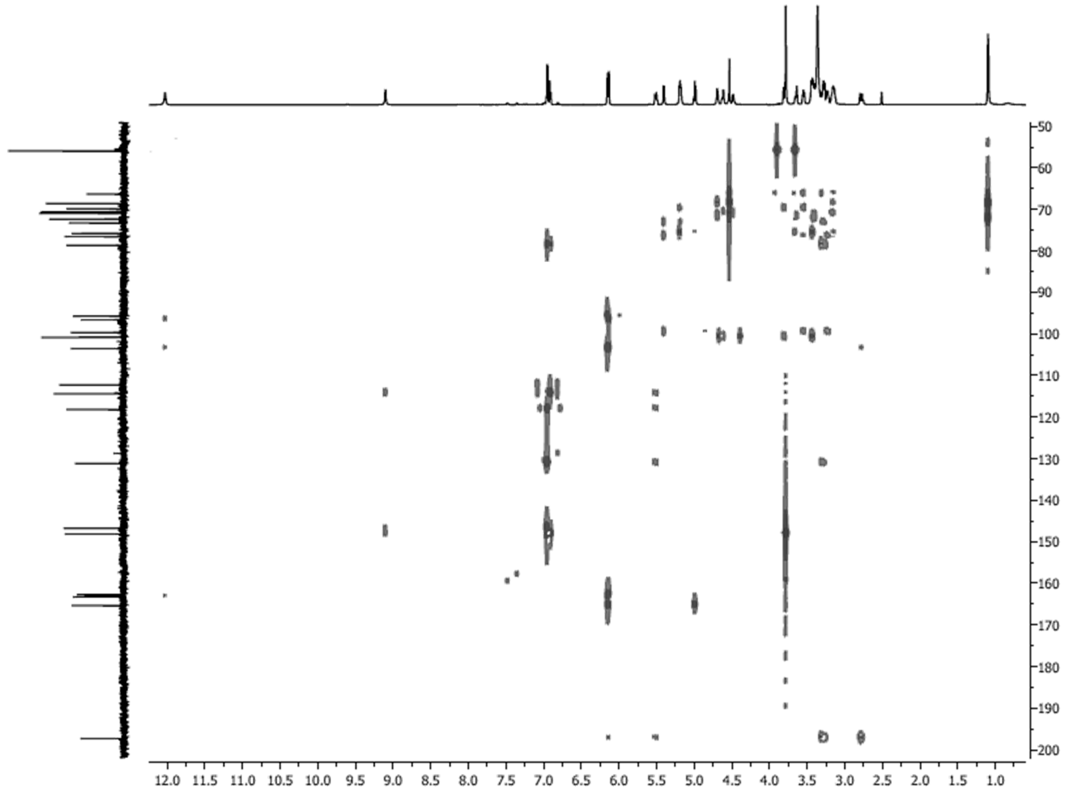

Figure S3. HMBC NMR data of obtained hesperidin (20 mg mL^{-1}) in DMSO-d_6 (2.50 ppm; 39.50 ppm) as a solvent, on Bruker *AVANCE* III 600 MHz equipment at 25 °C.

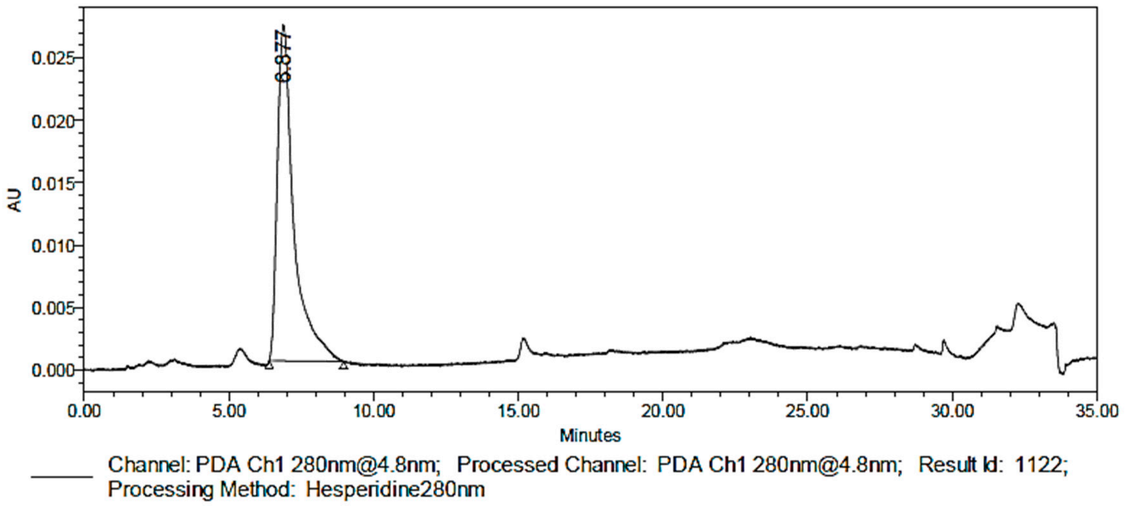

Figure S4. Chromatogram obtained from injection of obtained hesperidin sample in ultra-high-performance liquid chromatography (UHPLC) with reverse phase C18; the peak with the retention time at 6.877 min corresponds to hesperidin.

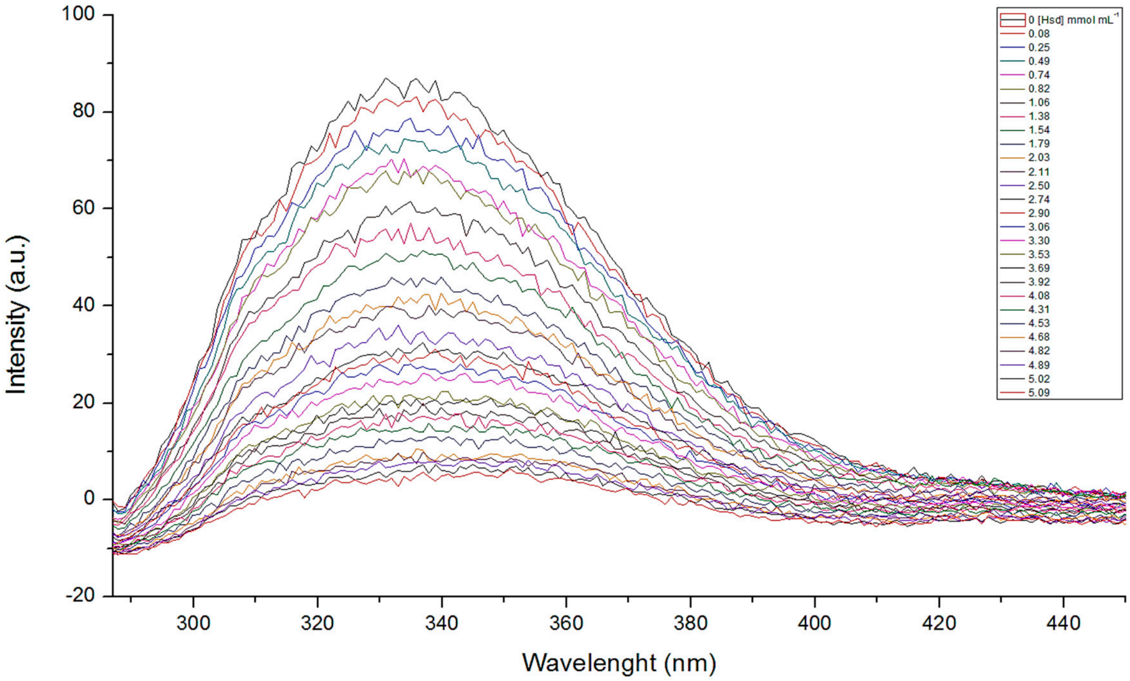

Figure S5. Fluorescence suppression - quenching effect of obtained hesperidin solution in DMSO (concentration from 0.00 to 5.09 mmol L^{-1}) on collagenase (*Clostridium histolyticum*, Sigma Aldrich) at 37 °C.

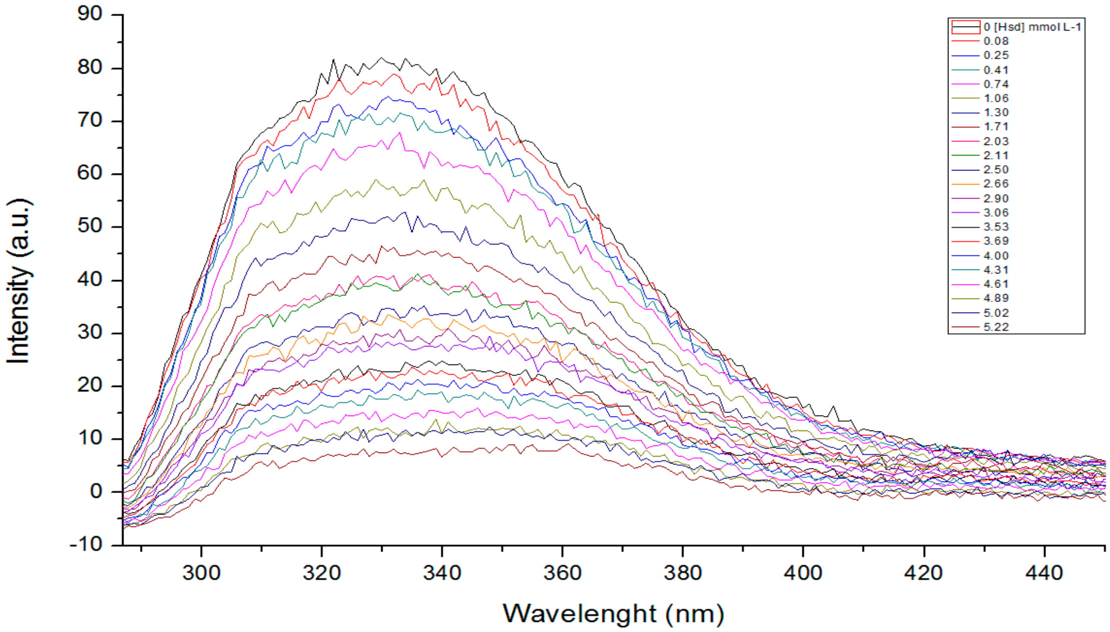

Figure S6. Fluorescence suppression - quenching effect of obtained hesperidin solution in DMSO (concentration from 0.00 to 5.22 mmol L^{-1}) on collagenase (*Clostridium histolyticum*, Sigma Aldrich) at 30 °C.
